# Isolation and Characterization of a Novel Facultative Anaerobic Filamentous Fungus from Japanese Rice Field Soil

**DOI:** 10.1155/2009/571383

**Published:** 2010-01-27

**Authors:** Akio Tonouchi

**Affiliations:** Faculty of Agriculture and Life Science, Hirosaki University, 3 Bunkyo-cho, Hirosaki, Aomori 036-8561, Japan

## Abstract

A novel filamentous fungus strain designated RB-1 was isolated into pure culture from Japanese rice field soil through an anaerobic role tube technique. The strain is a mitosporic fungus that grows in both aerobic and strict anaerobic conditions using various mono-, di-, tri-, and polysaccharides with acetate and ethanol productions. The amount of acetate produced was higher than that of ethanol in both aerobic and anaerobic cultures. The characteristic verrucose or punctuate conidia of RB-1 closely resembled those of some strains of the genus *Thermomyces*, a thermophilic or mesophilic anamorphic ascomycete. However, based on phylogenetic analysis with the small subunit (SSU) and large subunit (LSU) rDNA sequences, RB-1 was characterized as a member of the class Lecanoromycetes of the phylum Ascomycota. Currently, RB-1 is designated as an anamorphic ascomycete and is phylogenetically considered an *incertae sedis* within the class Lecanoromycetes.

## 1. Introduction

Rice fields are well known to be a major source of biological methane emission [1]. Many recent microbial analyses in rice fields have, therefore, focused on anaerobic microorganisms such as fermentative, acidogenic, and methanogenic microorganisms that are directly or indirectly involved in methane production [2]. Rice fields are usually flooded during the cropping season and drained after harvesting, and therefore, rice soils cycle through aerobic and anaerobic conditions throughout the year. During the drainage period, the major final decomposers of organic matter are aerobic or facultative anaerobic bacteria and filamentous fungi. Of these, many filamentous fungi are well known to secrete hydrolytic enzymes involved in biopolymer degradation, and therefore, they are considered to play significant roles in the decomposition of organic matter, especially in woods and upland soils. Filamentous fungi may also serve as decomposers of organic matter, such as plant debris and plant root exudation during the drainage period, in rice field soils. Kimura and Asakawa [3] showed the predominance of fungi in the microbial community of rice straw in a drained rice field using phospholipid fatty acids (PFLPs) as biomarkers. Hatamoto et al. [4] indicated by PCR-DGGE analysis that eukaryotes including fungi were associated with the decomposition of rice straw compost incorporated into rice soil especially at the midseason drainage. Many saprobic fungi have been isolated from Ukrainian rice soil before submerging and after draining [5]. However little is known about the ecological roles of such filamentous fungi during the water-logged phase because fungal species capable of anaerobic growth has not ever been isolated from rice soils.

Although most filamentous fungi are obligate aerobes, some obligate anaerobic species, collectively called rumen fungi and classified as Chytridiomycota [6], do exist in spite of the fact that their habitat is limited to the gastrointestinal tract of herbivorous mammals. Tabak and Cooke [7] reported anaerobiosis of 13 fungal strains isolated from oxygen-limited environments, such as sewage sludges, polluted waters, and organically enriched soils. Previously, Wada [8] reported that an unidentified spore-forming filamentous fungus colonized rice straws in submerged rice field soil where the anaerobic condition was sufficient for sulfate reducing. Saito et al. [9] showed that cellulose filter papers or cellophane films incorporated into submerged rice field soil were transiently colonized by fungal hyphae ten days after flooding. However, further studies on those fungal-like organisms were not conducted. Recently, it has been suggested that *Fusarium oxysporum* likely acquires energy for growth by denitrification [10] and by ammonia fermentation [11]. Fungal denitrification occurs in an O_2_ limited environment in which N_2_O is generated as a final denitrified product because of the lack of N_2_O reductase to generate N_2_ from N_2_O [10]. On the other hand, fungal ammonia fermentation occurs in an anaerobic environment in which NO_3_^−^ is reduced to NH_4_^+^ and ethanol is oxidized to acetate [11]. The fungal denitrification and ammonia fermentation in response to hypoxic conditions are considered to extensively distribute among many fungi [12]. These findings show the possibility that filamentous fungi capable of growth in the absence of oxygen are likely distributed in anaerobic environments in greater numbers than expected. These results suggest that some fungi capable of anaerobiosis certainly exist in rice soils. The author, therefore, attempted to isolate anaerobic filamentous fungi living in rice soils by applying the method of Hungate's roll-tube anaerobic technique [13] generally used in colonization of anaerobic prokaryotic cells. Here, the author reports the isolation and characterization of a novel facultative anaerobic fungus from Japanese rice field soil.

## 2. Materials and Methods

### 2.1. Fungal Strain

*Thermomyces lanuginosus* NBRC 9738 purchased from the National Institute of Technology and Evaluation was used for comparison with RB-1.

### 2.2. Media

Martin's medium [14] reduced with 0.03% cysteine hydrochloride was used for anaerobic fungal isolation from rice field soil. For usual cultivation, basal medium (0.1% KH_2_PO_4_, 0.05% MgSO_4_ 7H_2_O, 0.5% peptone, 0.1% yeast extract, and 1% glucose, (pH 7.0)) was used. For testing utilization of substrates, 1% glucose in the basal medium was substituted by the indicated substrates. For examining cultural and morphological characteristics, potato dextrose agar (PDA: NISSUI) was used. For anaerobic cultivation, media containing 0.0001% resazurine were prepared under an oxygen-free N_2_ atmosphere for visual check of reducing conditions. Before inoculation, the media were reduced by aseptically adding cysteine hydrochloride (adjusted to pH 7.0) to a final concentration of 0.03%. The tubes, vessels, and flasks used were closed with cotton plugs for aerobic cultivation and butyl rubber stoppers for anaerobic cultivation. If needed, suitable amounts of CaCO_3_ were added to the medium as a pH stabilizer to maintain the culture pH at 5.6–5.8. Media were solidified by adding 2% agar when required.

### 2.3. Isolation Procedure

Isolation of the fungus used in this study was essentially carried out in accordance with the Hungate roll tube technique [13]. All procedures except soil sampling and aerobic incubation on PDA during the final step of isolation were carried out anaerobically under a stream of oxygen-free N_2_. Soil was collected from 0–10 cm depth from the rice field at Kanagi farm of the Teaching and Research Center for Biocoexistence at Hirosaki University, Aomori, Japan, in April 2004. Some of the soil properties were as follows: pH (H_2_O), 6.0; water content, 38.9%. The soil sample was serially diluted in a 0.03% cysteine hydrochloride solution (pH 7.0) and transferred to agar-solidified medium (reduced with 0.03% cysteine hydrochloride) role tubes, which were then incubated at 25°C in a vertical position. After two months of incubation, the mycelium of a fungal colony formed on the agar was transferred to the same anaerobic fresh slant medium and incubated at 25°C. After two repetitions of the procedure, the mycelium developed on the anaerobic slant was transferred to a PDA plate and aerobically incubated at 25°C. A single spore isolate from the PDA plate culture was designated RB-1 and was used for further study. The cultures of RB-1 were maintained on PDA slants prepared in 25 mL tubes under both aerobic and anaerobic conditions. The RB-1 strain has been deposited in the Japan Collection of Microorganisms (JCM) as JCM13780.

### 2.4. Morphological Observations and Cultural Characteristics

For morphological observations and determination of cultural characteristics, the isolate was cultured aerobically or anaerobically on PDA plates at 25°C or on PDA thinly spread on a glass slide at 25°C (slide culture). AnaeroPack (Mitsubishi Gas Chemicals Co., Inc.) was used for generating anaerobic atmosphere. After staining with lactophenol cotton blue, the morphology of the isolate was observed under differential interference contrast with the use of an Olympus microscope (BX50F4) or under phase contrast with an Olympus microscope (MX50). For macroscopic observations, a stereomicroscope (Olympus SZH10) was used. Colony colors were evaluated based on the Methuen Handbook of Color [15].

### 2.5. Phylogenetic Analysis

Genomic DNA of the isolate was prepared as described previously [16]. Nearly full-length small subunit (SSU) rDNA (1,760 bp) was PCR-amplified using the primer pair E21f (5′-ATCTGGTTGATCCTGCCAGT-3′) and E1778r (5′-AATGATCCTTCCGCAGGTTC-3′) [17]. For amplification of the internal transcribed spacer (ITS) region and the 5′ end of large subunit (LSU) rDNA including the D1-D2 domains (ITS-LSU rDNA) (1,259 bp), a primer set ITS5 (5′-GGAAGTAAAAGTCGTAACAAGG-3′) [18] and NL4 (5′-GGTCCGTGTTTCAAGACGG-3′) [19] was used. The PCR products were directly sequenced using the Thermo Sequenase CY5.5 dye terminator cycle sequencing kit and the SEQ 4.4 personal sequencing system (Amersham). The sequences of SSU rDNA and ITS-LSU rDNA of the isolate are deposited in the DDBJ/GenBank/EMBL databases under the accession numbers AB246879 and AB517730, respectively. The determined sequences and those of relatively closely related taxa to the isolate retrieved from the DDBJ/GenBank/EMBL databases by searching with BLAST program were aligned using the CLUSTAL W program [20]. The evolutionary distances between the sequences were calculated using Kimura's two-parameter model [21], and the phylogenetic trees were constructed by the neighbor-joining method [22] using the MEGA 4.0 [23] computer program. Bootstrap analyses were conducted to assess the confidence limits of the branching by 1000 heuristic replicates.

### 2.6. Biophysical and Biochemical Characteristics

All anaerobic incubations were carried out under an oxygen-free N_2_ atmosphere. The temperature and pH profiles of the isolate were determined by measuring the diameters of the colonies developed under aerobic conditions. The temperature profile was tested on the PDA plate by incubating at 4–50°C. The pH profile was tested on the basal medium plate at a pH range from 2.0 to 9.5. Incubations were carried out at 30°C.

For testing available substrates for fermentation, autoclaved or filter-sterilized substrates were added to 10 mL of basal medium instead of glucose in culture tubes, and the mycelium from the PDA slant was inoculated into the medium. Incubations were carried out at 30°C for up to 3 months under aerobic or anaerobic conditions without shaking. The substrates were considered positive when acetate and ethanol were detected in the medium. Ethanol and acetate were measured as mentioned in Analytical Procedures.

Time course of growth by the isolate under aerobic and anaerobic conditions was determined using 500 mL Erlenmeyer flasks with 100 mL of basal medium inoculated with the equivalent of 1.5 mg (dry weight) mycelium previously grown aerobically in 10 mL basal medium at 30°C for 10 days. After inoculation, the flasks were incubated at 30°C in a reciprocal shaker operating at 100 rpm. Samples were collected at various intervals by filtration through nitrocellulose filters and were measured after drying to a constant weight at 105°C; the resulting filtrates were used to determine the residual glucose and fermentation products.

Growth yields of the isolate on aerobic and anaerobic medium were determined using 125 mL serum bottles with 100 mL of basal medium inoculated with a trace amount (<0.1 mg) of mycelium from the PDA slant picked with a needle tip. After static incubation at 30°C for 10 days, cultures underwent the same analytical procedures for growth analysis as described above.

Growth ability of the isolate under anaerobic conditions was confirmed by a successive transfer experiment as described by Gleason and Gordon [24]. The mycelium of the isolate grown on the PDA slant was inoculated into 100 mL anaerobic basal medium prepared in a 125 mL serum bottle. The culture bottle was incubated at 30°C and a small part of the mycelium grown in the medium was transferred every five days into the same medium. Exposure of mycelium to an atmospheric condition was avoided as possible with an immediate transfer to the next fresh medium.

All experiments were repeated more than three times to confirm reproducibility.

### 2.7. Analytical Procedures

Glucose was quantified using the anthrone-sulfuric acid method [25]. Ethanol was analyzed in a gas chromatograph (Shimadzu GC8A) equipped with an FID detector and a Porapaq N column (Waters). Organic acids were measured by using HPLC (Organic Acid Analysis System, Shimadzu) equipped with an ion exclusion column (Shim-pack SCR-101H) and detected by a post-column pH-buffered electroconductivity detection method [26] according to the manufacturer's instructions. Glycerol was enzymatically assayed with glycerokinase using commercial kits (Boehringer Mannheim). Gas samples were determined by gas chromatography (Shimadzu GC8A) equipped with a TCD detector and a WG-100 column (GL Sciences).

## 3. Results

### 3.1. Morphological and Cultural Characteristics

**Figure 1:**
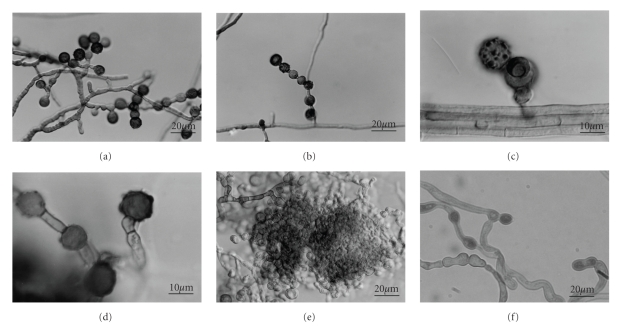
Morphology of RB-1 grown on PDA under aerobic conditions: (a)–(e) as viewed by differential interference contrast microscopy, and under anaerobic conditions (f) as viewed by phase contrast microscopy. (a) Alleurio conidia and conidiogenous cells; (b) formation of chained conidia; (c) a verrucose surface structure of the conidium; (d) conidia and chlamydospores; (e) a structure indicative of some primordia; and (f) conidia- and chlamydospore-like structures of an anaerobically grown culture.

The strain designated RB-1 was a filamentous fungus isolated from Japanese rice field soil that could grow under both aerobic and anaerobic conditions. After six weeks of aerobic incubation at 25°C on a PDA plate, the colonies were olive to olive-green in color, with a floccose texture, and few aerial mycelia. The color of the colonies was white initially and then turned from light green to dark green and eventually from olive to olive-green. Exudates and soluble pigment were not produced. In the vegetative stage, septate hyphae, which were 2.0–3.0 *μ*m in diameter, changed in color from hyaline to bright brown with aging and were formed in the agar and on the agar surface. The morphological features of RB-1 after two months of aerobic incubation are shown in Figures 1(a)–1(e). Distinct conidiophore-like structures were absent (Figure 1(a)). Conidiogenous cells were cylindrical or indistinguishable from the vegetative hyphae (Figure 1(a)). Single or occasionally chained aleurioconidia, which were hyaline when young and brown to dark brown when mature, were borne on the tips of the slightly swollen conidiogenous cells or directly on the sides of the vegetative hyphae (Figures 1(a) and 1(b)). Mature conidia were globose-shaped or sometimes slightly subglobose-shaped single cells, 7.5–11.3 *μ*m in diameter, and their surfaces were verrucose or punctuate (Figure 1(c)). Chlamydospores, which were smooth or verrucose, globose to oval, light brown to dark brown, and 9.0–11.0 *μ*m in diameter, occurred frequently in the vegetative hyphae (Figure 1(d)). Sexual reproductive organ development was not observed. Instead, primordium-like structures constructed with subglobose and globose cells were formed after two months of incubation (Figure 1(e)).

Colonies on the anaerobic PDA plate were always white, and aerial mycelia were rarely observed, at least during the incubation period (up to 3 months). In the presence of CaCO_3_, colonies on the anaerobic PDA plate developed at a rate similar to that under aerobic conditions with dissolving insoluble particles of CaCO_3_. However, in the absence of CaCO_3_, colony development was slower than that under aerobic conditions and ceased when the colonies reached approximately 60 mm in diameter, which indicates that the lowered pH caused by acid production inhibited colony development. Although normal conidium development did not occur under any anaerobic conditions, some swollen cells looking little similar to conidium and chlamydospore structures were observed after three months of incubation (Figure 1(f)).

*Thermomyces lanuginosus* [14], a fungal species apparently related morphologically to RB-1, did not grow under any anaerobic conditions. Moreover, acid production in its aerobic cultures was not observed different from those of RB-1. Hence further physiological and biochemical studies on *T. lanuginosus* were not carried out.

### 3.2. Phylogenetic Analysis

**Figure 2:**
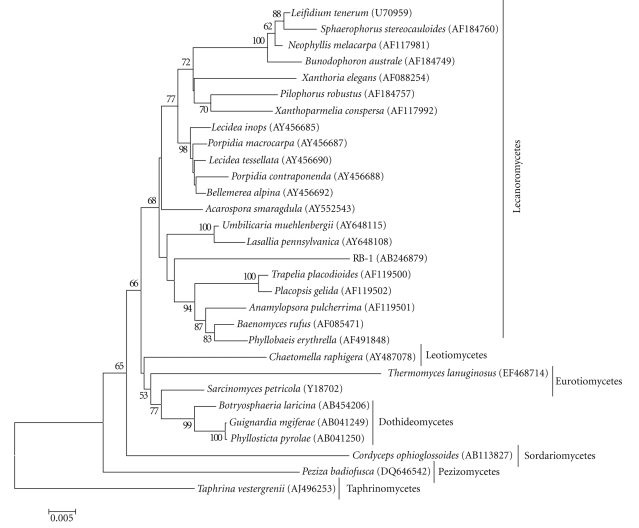
Phylogenetic tree for RB-1 constructed by the neighbor-joining method on the basis of the SSU rDNA sequences retrieved from the DDBJ/GenBank/EMBL databases. DDBJ/GenBank/EMBL accession numbers of retrieved sequences are indicated in parentheses. The Bootstrap values based on 1000 replications are given on the nodes as percentages above 50%. *Taphrina vestergrenii* was used as the outgroup. Bar: 0.005 substitutions per nucleotide position.

**Figure 3:**
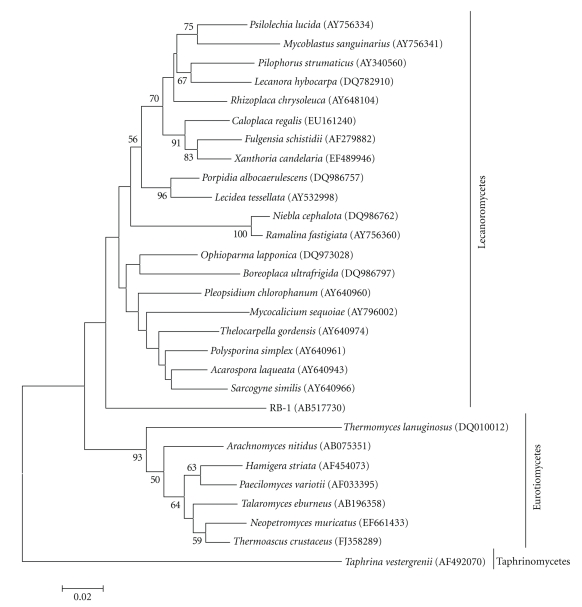
Phylogenetic tree for RB-1 constructed by the neighbor-joining method on the basis of the D1-D2 sequences retrieved from the DDBJ/GenBank/EMBL databases. DDBJ/GenBank/EMBL accession numbers of retrieved sequences are indicated in parentheses. The Bootstrap values based on 1000 replications are given on the nodes as percentages above 50%. *Taphrina vestergrenii* was used as the outgroup. Bar: 0.02 substitutions per nucleotide position.

The SSU rDNA sequence of RB-1 showed less than 96% similarity with those of other known fungal species and was most closely related to *Bellemerea alpina* (95.3% identity), which belongs to the order Lecanorales of the class Lecanoromycetes. A phylogenetic tree based on the sequences of SSU rDNA retrieved from the DDBJ/GenBank/EMBL databases is shown in Figure 2. Totals of 30 taxa (1,659 to 1,669 bp after alignment) were used. In this tree, RB-1 is placed within the class Lecanoromycetes of the phylum Ascomycota. *T. lanuginosus* showed a relatively low SSU rDNA sequence similarity (92.0% identity) with RB-1 and the presence in the class Eurotiomycetes (Figure 2). In order to gain further insight into the classification of RB-1, a phylogenetic analysis using the LSU rDNA D1-D2 sequences was conducted. A DDBJ/GenBank/EMBL search revealed that the D1-D2 sequence of RB-1 had low similarity with those of related fungal species (less than 89% identity). The D1-D2 sequence of *Caloplaca regalis*, which is classified as Teloschistales of the class Lecanoromycetes, showed the highest similarity (88.6% identity) with that of RB-1. A phylogenetic tree constructed based on the D1-D2 sequences, which were retrieved from the DDBJ/GenBank/EMBL databases, is shown in Figure 3. Totals of 29 taxa (496 to 500 bp after alignment) were used to construct a phylogenetic tree. In this tree, RB-1 is related to Lecanoromycetes rather than to Eurotiomycetes. *T. lanuginosus* is placed in the class Eurotiomycetes as same as the phylogenetic tree constructed with the SSU rDNA sequences (Figure 2). The identity of the D1-D2 sequences between RB-1 and* T. lanuginosus *was 82.1%.

Phylogenetic analyses using the ITS1 and ITS2 sequences were not carried out because ITS sequences related to those of RB-1 which are suitable for construction of phylogenetic trees with high confidence could not be retrieved from the DDBJ/GenBank/EMBL databases.

### 3.3. Physiological and Biochemical Characteristics

**Figure 4:**
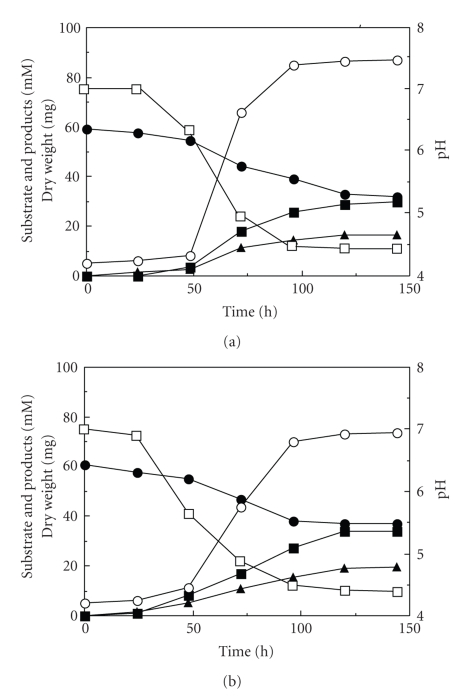
Growth of RB-1 in both aerobic (a) and anaerobic mediums (b). RB-1 was grown on glucose in basal medium with 1% glucose as described in Section 2. Symbols: (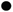) glucose; (■) acetate; (▲) ethanol; (*○*) dry weight; and (□) pH. The data shown are from representative experiments of four independent experiments with similar results.

Physiological and biochemical properties are summarized in Table 1. RB-1 grew by consuming glucose and producing acetate and ethanol as fermentation products under both aerobic (Figure 4(a)) and anaerobic (Figure 4(b)) conditions. Growth rate was almost similar between the aerobic and anaerobic cultures. Other possible products including secondary organic compounds, such as hydrogen, succinate, pyruvate, formate, citrate, lactate, glycerol, isopropanol, acetone, propionate, butyrate, glyoxalate, and malonate, were not detected. Growth and substrate consumption terminated immediately after the pH dropped below ~4.7. Glucose was completely consumed in both aerobic and anaerobic cultures and growth was not terminated if the pH of the cultures was stabilized with CaCO_3_; acetate and ethanol produced in the pH-stabilized aerobic culture were consumed immediately after the exhaustion of glucose (data not shown).

The available initial pH for the growth of RB-1 ranged between 2.5 and 8.0 with an optimum pH of 5.0–6.0. Only slight growth was observed at pH 8.0. No growth occurred at pH 9.0 and 2.0. RB-1 grew at temperatures between 4 and 37°C, with an optimum around 25–30°C. At 37°C, little increase in colony diameter occurred. No growth occurred at 40°C or above. Conidium development was more prolific at 25°C. In both static aerobic and anaerobic liquid cultures, RB-1 always grew as a spherical cotton ball on the bottom of the flasks or tubes but not as a floating mat or pellicle.

RB-1 could use D-glucose, D-galactose, D-mannose, D-fructose, D-xylose, L-arabinose, sucrose, maltose, cellobiose, trehalose, raffinose, starch, inulin, xylan, and pectin as fermentation and growth substrates. Fermentation on trehalose, inulin, and xylan was much weaker than that on other available substrates. All these substrates supported growth and were fermented to acetate, ethanol, and carbon dioxide. In the aerobic cultures, all substrates that were fermentable under anaerobic conditions were also converted to ethanol and acetate. On the other hand, RB-1 did not use L-rhamnose, lactose, melibiose, D-mannitol, D-sorbitol, glycerol, Avicell, carboxymethylcellulose, or chitin as fermentation and growth substrates. Growth yield and quantitative determinations of ethanol and acetate production on D-glucose are given in Table 2. More acetate than ethanol was produced under both anaerobic (1.8 fold) and aerobic (1.9 fold) conditions.

Growth of RB-1 under anaerobic conditions (in a cysteine-reduced liquid medium) was sustained constantly from the initial culture after eleven successive transfers in the same medium.

## 4. Discussion

Because filamentous fungi are commonly recognized as obligate aerobic organisms, little interest has been paid to their ecological roles and functions in anaerobic environments, including flooded rice field soils. Accordingly, filamentous fungi have been considered to be less important constituents of natural anaerobic ecosystems; few studies on anaerobic filamentous fungi in natural habitats have been reported [7, 27–29]. In the present study, the author reports on the isolation of a novel facultative anaerobic filamentous fungus from Japanese rice field soil.

Since no sexual reproductive organs were observed, from a morphological standpoint, RB-1 is currently considered a mitosporic fungus. The morphological characteristics of RB-1 are closely related to those of *Thermomyces lanuginosus* and *Thermomyces verrucose* [14]; the former species has also been isolated from Japanese field soil [30]. However, these species differ from RB-1 in forming single conidia from the conidiophores and their temperature profiles [31]. Moreover, *T. lanuginosus* showed no anaerobic growth as reported previously [32]. The phylogenetic statuses of *T. lanuginosus* and *T. verrucose* are indistinct, so they are currently classified as mitosporic ascomycetes (anamorphic ascomycetes) [33]. However, recently it has been shown that *T. lanuginosus* is placed in the class Eurotiomycetes and in the order Eurotiales based on the phylogenetic analysis using SSU rDNA and ITS sequences [34] as shown here (Figures 2 and 3). Usually, in mitosporic fungi, similarities in morphological characteristics do not necessarily indicate phylogenetic relations. Certainly, there are relatively low similarities of the SSU rDNA (92.0% identity) and the D1-D2 sequences (82.1% identity) between RB-1 and *T. lanuginosus*, which indicates that they are phylogenetically distant. According to the phylogenetic analysis based on the SSU rDNA and LSU rDNA sequences, RB-1 was probably placed within the class Lecanoromycetes of Ascomycota (Figures 2 and 3) although the precise phylogenetic position is currently unclear. Further phylogenetic studies using other markers such as elongation factors and mitochondrial rDNA should be required. Thus, although RB-1 is probably an anamorph of an unknown ascomycete considered an *incertae sedis* within the class Lecanoromycetes, the classification is still inadequate to define its precise taxonomic status; therefore, further detailed taxonomic analyses will be needed for nomenclature.

RB-1 can grow both aerobically and anaerobically, unlike most other ethanol- and acetate-producing fungi; almost all of which can ferment but not grow under anaerobic conditions [35, 36]. In successive transfer experiments for confirming the anaerobiosis, the growth ability of RB-1 in the strict anaerobic liquid medium reduced with cysteine was steadily maintained even after eleven successive transfers. A study of anaerobic growth of zygomycetes showed that strains that are apparently capable of anaerobic growth, but require extremely small amounts of oxygen for any growth, are diluted out during successive transfers in anaerobic medium, whereas a strain truly capable of anaerobic proliferation continues growth after ten successive transfers [24]. Consequently, it is concluded that RB-1 is a facultative anaerobic fungus. As far as known to the author, few facultative anaerobic filamentous “higher fungi” that can produce metabolic energy by simple fermentation, such as the yeast *Saccharomyces cerevisiae*, have been identified [28, 29]. As shown in Figure 4 and Table 2, the amount of acetate was higher than that of ethanol, unlike other fermentable ethanol-producing fungi; almost all of which produce smaller amounts of acetate than ethanol [36]. A simple comparison cannot be made because of differences in the culture conditions and media between those previous studies and this study; hence the reason for this remains unclear, and metabolic analyses of RB-1 are ongoing. RB-1 produced significant amounts of ethanol and acetate under complete aerobic conditions. This phenomenon is similar to that of the Crabtree-positive yeast cultures in which ethanol production occurs under full aerobic conditions when sugar is present in excess [37, 38]. Hence, RB-1 is probably a Crabtree-positive organism. Productions of possible compounds other than ethanol, acetate, and carbon dioxide in the culture of RB-1 were not detected. If produced, their amounts may be smaller than the detection limits (<ca. 0.1–0.5 mM) of measuring systems (HPLC and GC) used in this study. Interestingly, RB-1 shows a significant acetate kinase (ACK) activity, thought to be found strictly in prokaryotic cells, during the growth under both aerobic and anaerobic conditions (unpublished data). Recently the presence of the ACK genes in several eukaryotes inducing fungi was identified [39]. However, the activity of ACK from those eukaryotes has not been confirmed. Thus, RB-1 may be an interesting organism for study of acetate metabolism. The author and coworkers are now attempting to purify ACK from RB-1.

The phylogenetic, biophysical, and biochemical features of RB-1 indicated here can be distinguished from those of other known fungal species. Especially, RB-1 is phylogenetically far from known fungal genera. Hence, the author concludes that RB-1 is a novel fungus. 

As rice fields that are usually exposed to rotating periods of irrigation and drainage during cropping, plowed soils of the fields also rotate through anaerobic and aerobic conditions. Accordingly, microbial colonies will go through changes responsive to irrigation (anaerobic conditions) and drainage (aerobic conditions). Moreover, plowed soils of flooded rice fields are heterogeneous environments in which oxygen concentrations differ from those of other anaerobic sites. For example, bulk soils below the soil-water interface are strictly anaerobic; because oxygen is supplied through floodwaters and the aerenchyma of rice plants, the soil surface, the rhizosphere, and the rhizoplane are aerobic, respectively [40]. Facultative anaerobes are probably more adaptable microorganisms in such atypical sites compared to obligatory aerobic or anaerobic microorganisms. Hence, although the ecological role and function of RB-1 in rice soil ecosystem are currently unknown, the abilities of RB-1 to acquire metabolic energy and to proliferate in both aerobic and anaerobic environments may be advantageous in such environments. Previously, Wada [8] reported that an unidentified spore-forming filamentous fungus colonized rice straws in submerged rice soil where the anaerobic condition was sufficient for sulfate reducing. Recently, several researchers reported that when nitrate is supplied, many filamentous fungi can acquire metabolic energy in oxygen-limited and anaerobic environments by nitrate respiration and ammonia fermentation, respectively [10, 11, 41]. A recent research indicated that up to 89% of N_2_O emitted from soils to atmospheres could be attributed to fungal activity [42]. All these findings suggest that filamentous fungi capable of growth in the absence of oxygen are likely distributed in anaerobic environments in greater numbers than expected.

## Figures and Tables

**Table 1:** Physiological and biochemical properties of RB-1.

Characteristic	
pH range (optimum)	2.5–8.0 (5.0–6.0)
Temperature range (optimum)	4–37°C (25–30°C)
Fermentation and growth substrate	
D-Glucose	+
D-Galactose	+
D-Mannose	+
D-Fructose	+
L-Rhamnose	−
D-Xylose	+
L-Arabinose	+
Sucrose	+
Maltose	+
Cellobiose	+
Melibiose	−
Lactose	−
Trehalose	+(weak)
Raffinose	+
Strach	+
Avicell	−
Carboxymethylcellulose	−
Inulin	+(weak)
Xylan	+(weak)
Pectin	+
Chitin	−
D-Mannitol	−
D-Sorbitol	−
Glycerol	−

**Table 2:** Growth yield and yields of ethanol and acetate from growth of RB-1^*a*^.

Growth conditions	Yield (mol/100 mol of glucose)		Grwoth yield (g dry weight/mol of glucose)
	Acetate	Ethanol	
Anaerobic	148.0 ± 11.2	84.4 ± 5.4	3.3 ± 0.5
Aerobic	149.5 ± 9.9	77.8 ± 7.3	4.6 ± 0.4

^a^Values are means ± SD of tripricate experiments.
